# Adding 11C-acetate to 18F-FDG at PET Examination Has an Incremental Value in the Diagnosis of Hepatocellular Carcinoma

**DOI:** 10.4274/Mirt.87

**Published:** 2012-04-01

**Authors:** Patricia Larsson, Dag Arvidsson, Mikael Björnstedt, Bengt Isaksson, Ulf Jersenius, Hooman Motarjemi, Hans Jacobsson

**Affiliations:** 1 Karolinska University Hospital Solna, Departments of Radiology and Nuclear Medicine, Solna, Sweden; 2 Karolinska University Hospital Solna, Department of Surgery, Stockholm, Sweden; 3 Karolinska University Hospital Huddinge, Department of Pathology, Huddinge, Sweden; 4 Karolinska University Hospital Huddinge, Department of Surgical Gastroenterology, Huddinge, Sweden

**Keywords:** (11C)-acetate, 18F-FDG, Hepatocellular carcinoma, tomography, positron-emission, sensitivity

## Abstract

**Objective:** The sensitivity of FDG at PET examination of Hepatocellular Carcinoma (HCC) is restricted. In a few studies, all done in Oriental patients, PET-examination with ^11^C-acetate has shown a higher accuracy than with FDG. In the current study, the uptake of ^11^C-acetate has been compared with the uptake of FDG in the primary HCC in a cohort of Occidental patients.

**Material and Methods:** 44 patients underwent PET-examination with both tracers with a mean of 9 days between the examinations. 26 patients had a microscopical diagnosis and 18 were diagnosed with multimodal radiological methods. At least one relevant radiological examination was available for comparison.

**Results:** At visual evaluation, 13 of the HCC’s were positive at PET-examination using FDG and 34 were positive using ^11^C-acetate (p<0.001). Median tumor SUV_mean_ of ^11^C-acetate was 4.7 and of FDG was 1.9 (p<0.001). There was also a higher uptake of ^11^C-acetate by the surrounding liver tissue than of FDG. Median liver SUV_mean_ of [u]11[/u]C-acetate was 3.2 and of FDG it was 1.7 (p<0.001). This corresponded to a median tumour/liver tissue ratio for ^11^C-acetate of 1.4 and for FDG of 1.0 (p<0.05). Previous reports of a negative correlation between the uptake of the tracers were weakly supported. In 4 large tumors some portions being hot using one of the tracers were cold using the other tracer and vice versa.

**Conclusion:** Adding registration with ^11^C-acetate to registration with FDG at PET-examination has an incremental value in the diagnosis of HCC. A higher tumor uptake of ^11^C-acetate cannot be taken full advantage of because of a higher uptake also by the surrounding liver tissue.

**Conflict of interest:**None declared.

## INTRODUCTION

Positron emission tomography (PET) has become a powerful tool for tumor detection, staging and therapy evaluation. The common tracer for this purpose is 18F-2-fluoro-2-deoxy-D-glucose (FDG), while there are certain tumors in which the value of this tracer is restricted. This is the case in Hepatocellular Carcinoma (HCC) in which a sensitivity by FDG of 40-50% has been reported (1,2,3). The low sensitivity is considered to be due to a high glucose-6-phosphatase activity in normal liver cells, which leads to an extrusion of the tracer, a mechanism that is believed to be more or less retained in HCC (1).

HCC is a complication in patients with liver cirrhosis and a common malignancy in Asian countries. With an increase of hepatitis C infection together with alcohol abuse, HCC shows an upward trend also in the Occident (4,5). In a study from Hong Kong in 2003, it was reported that the combination of PET examinations with FDG and ^11^C-acetate has an incremental value when compared to single-tracer imaging in the diagnosis of HCC (6). We early adopted the dual-tracer PET technique as a routine procedure in patients with suspected HCC. In this retrospective study, the two tracers have been evaluated in the diagnosis of the primary HCC. The background being the number of studies in this field is small, some are based on a restricted number of observations and all studies are done in Asian patients. SUV_mean_ and SUV_max_ of the tumors were calculated and also compared with the activity of the surrounding regular liver tissue as well as with the blood activity of the large mediastinal vessels.

## MATERIALS AND METHODS

**Patients**

Sixty-four patients with suspected HCC having undergone PET examination both with FDG and ^11^C-acetate were reviewed. Of these, 44 were clear-cut (no other malignancy and no previous liver surgery, lipiodol treatment or chemotherapy) and included in the study. This was approved by the regional Ethical committee. There were 28 males and 16 females. Their mean age was 65 years (range 24−85). The ethnical background was established as far as possible. 42 patients were of Occidental origin and 2 patients originated from an Asian country. 26 patients had a microscopical diagnosis and 18 were diagnosed with multimodal radiological methods according to The European Association for the Study of the Liver (**7**) together with clinical presentation.

Sixteen of the patients were positive for hepatitis C. Of these, 2 also had hepatitis B and 5 abused alcohol. 2 patients were positive only for hepatitis B. 5 patients had alcohol abuse as the only obvious reason for cirrhosis. There was 1 patient with hemochromatosis, 2 with porphyria, 1 with Crohn´s disease, 1 with primary biliary cirrhosis. 3 patients had cirrhosis of unknown cause. 13 patients had no known liver disease.

The tumor size could be assessed from the radiological examinations in 42 patients (Table 1).

**Histopathological Evaluation**

Twenty-four patients were diagnosed with histopathology (biopsy or resected specimen), one with fine needle aspiration cytology, and one later on at autopsy. The histological slides were blindly re-evaluated and the degree of differentiation was assessed by MB. Five tumors were poorly differentiated, 14 were moderately differentiated and 5 were well differentiated. 

**PET Examination**

An ECAT EXACT 31 PET camera (CTI, Knoxville, Tenn., USA) was used. PET registration (acquisition?) with ^11^C-acetate was initiated 10 min after i.v. administration of 600 MBq of the tracer. PET registration (acquisition?) with FDG was initiated after fasting for at least 4 h and, a rest period of 1 hour, and 60 min after i.v. administration of 400 MBq of the tracer. There was a mean of 9 days between the two examinations. A series of consecutive 10-min scan from the inguinal region cranially including the chest was acquired. Images were reconstructed using the commercial iterative OSEM algorithm. Correction for attenuation and photon scattering was made using transmission maps obtained from 3 ^68^Ge/^68^Ga rods located around the patient applying the camera software.

**Evaluation of PET Examinations**

Evaluation of both studies in the patient was made at the same occasion by PL using a Siemens Leonardo viewing station after pairwise aligning the examinations. In each patient, one liver lesion was assessed. In cases of biopsy and multiple liver tumors, the lesion subjected to biopsy was assessed. In all patients, at least one radiological examination (out of CT and MRI) was always compared.

Identical 3D isocontour-ROI’s were used to assess SUV_mean_ and SUV_max_ of the lesions and SUV_mean_ of the background tissues (surrounding/non malignant liver tissue and the large mediastinal vessels representing the blood activity; mediastinal activity) of both examinations. In most lesions, a cut-off of 50% of SUV_max_ was adequate. In a few examinations with low lesion to background ratio or in lesions with heavily necrotic centres, cut-off adjustments was made. In patients where the lesion showed no or a very slight uptake with one tracer, but considerable uptake with the other tracer, the ROI was transferred between the examinations. In one examination with FDG, SUV calculations could for technical reasons not be achieved while visual evaluation was possible. The lesions were also characterised as being visually positive or negative with regard to the tracers. Tumours showing a reduced tracer accumulation compared to the surrounding regular liver activity were considered negative.

**Statistical Methods**

Categorical data were summarised using frequency counts and percentages. Continuous data were presented as median and range (minimum-maximum) or inter-quartile range (P25-P75). To evaluate the difference in tumor positivity between the two tracers at visual assessment McNemar’s test was used. The Sign test was performed for comparison between the two agents at the SUV-calculations. The association between uptake of ^11^C-acetate and FDG was first inspected by linear regression analysis. Residual analysis revealed deviation from linearity why? Spearman Rank correlation coefficients were calculated. As no trend at the various evaluations was assumed, two-sided p-levels were always calculated. p <0.05 was considered significant. 

## RESULTS

Thirteen of the 44 primary HCC’s were positive at PET examination with FDG and 34 were positive at examination with ^11^C-acetate. 8 tumors were positive for both radiopharmaceuticals (Table 2). Adding examination with ^11^C-acetate to examination with FDG significantly increased the detectability of HCC (p<0.001). Still, 5 patients were negative using both tracers.

**Figure 1:**
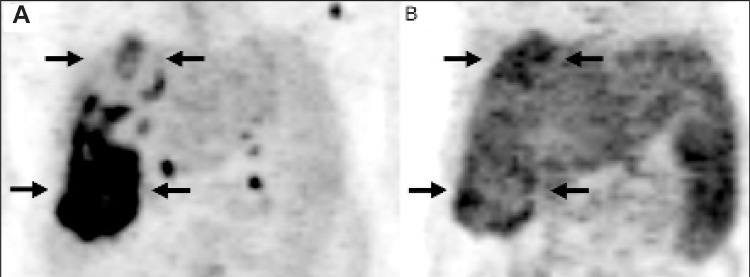
PET examinations (A-P MIP’s) with FDG (A) and 11C-acetate (B) ina 58-year old woman with a large HCC (arrows). This illustrates theparadoxical effect of the higher uptake of ^11^C-acetate by the surroundingliver tissue compared to the uptake of FDG. In this patient, the tumorSUVmean was 3.2 for both tracers. SUVmean for FDG of surrounding liver tissue (A)was 1.1 and SUVmean for ^11^C-acetate (B) was 2.6. Thiscorresponded to tumor-to-background ratios of 3.0 and 1.2, respectively

The mean tumor uptake of ^11^C-acetate in all patients was higher than the corresponding uptake of FDG (Table 3). Since the mediastinal (blood) activity did not much differ between the tracers, the ratio between the tumor activity and the blood activity of ^11^C-acetate was also higher than the corresponding ratio of FDG. The absolute uptake of ^11^C-acetate by the liver tissue surrounding the tumor was higher than the corresponding uptake of FDG. The net effect of this was that in the ^11^C-acetate positive examinations, the contrast between the tumor activity and the surrounding liver activity was lower than the corresponding ratio of the FDG positive tumors(Table 4, Figure 1).

The correlation between the uptake of the two tracers was analysed in all evaluable patients and separately in the 8 patients being positive for both agents (Table 5). All evaluations showed a negative, but non-significant, tendency (Spearman Rank correlation coefficient <1.0).

**Figure 2:**
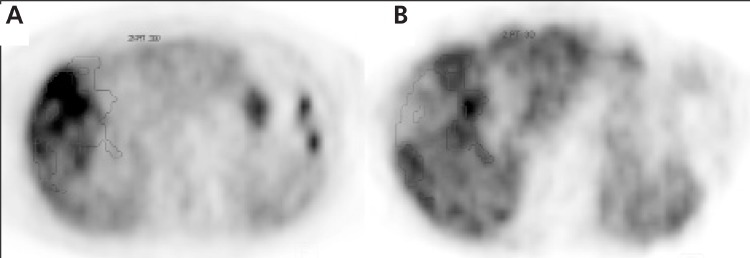
Transversal sections at the same level from PET examinationswith FDG (A) and ^11^C-acetate (B) in a 76-year old woman with a large HCCdiagnosed according to the Barcelona criteria. The figure illustrates adiscordant uptake between the two tracers. Red delineates the 50% isocontourlevel of FDG and blue delineates the 50% isocontour level of ^11^C-acetate

**Figure 3:**
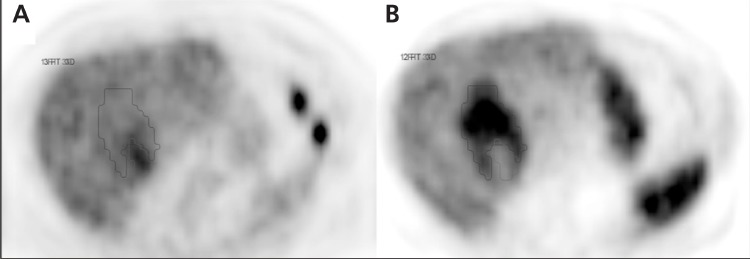
Transversal sections at the same level from PET examinationswith FDG (A) and ^11^C-acetate (B) in a 70-year old man with a large, well differentiatedHCC confirmed at histopathology after surgical resection.The figure illustrates a discordant uptake between the two tracers. Reddelineates the 50% isocontour level of FDG and blue delineates the 50%isocontour level of ^11^C-acetate

In 4 of the 8 patients where the tumor accumulated both tracers, there was a striking disconcordance of the activity distribution within the same tumor. Portions being hot using one of the tracers were relatively colder using the other tracer and vice versa (Figures 2 and 3). In the other 4 patients, the uptake of the tracers was concordant. All tumors showing a disconcordant uptake pattern were large; mean diameter 9 cm.

## DISCUSSION

In the first study of HCC and ^11^C-acetate, from Hong Kong, adding examination with this tracer to PET examination with FDG increased the detection? sensitivity of HCC. It was also reported that highly differentiated HCC is better depicted by ^11^C-acetate and poorly differentiated types are better depicted by FDG (6). Similar findings were later reported in the evaluation of HCC metastases (8). A study from Korea confirmed that the addition of ^11^C-acetate to FDG at PET examination increases the overall sensitivity for the detection of HCC. ^11^C-acetate accumulation was also higher than FDG accumulation in well-differentiated HCC (9). Another study from Korea reported that when HCC showed a low FDG uptake it was ^11^C-acetate-avid, and vice versa (10). Similar findings have been reported in an animal study (11). Studies using only FDG have shown that tracer uptake is higher in poorly differentiated HCC than in well-differentiated tumors (3,12,13). All previous studies have been made in Asian populations where the incidence of Hepatitis B and C is high and accounts for ?80% of the etiology of HCC (14). The current study was made in mainly Occidental patients of which ?40% of the patients had viral hepatitis and alcoholic cirrhosis is a more common risk factor for HCC. Since the etiology may affect the functional characteristics of the tumor (15), the current study may be regarded complementary to previous reports.

Because of a substantial risk of needle track seeding of tumor cells, biopsy or cytology is often avoided in suspected HCC (16). The European Association for the Study of the Liver has proposed that in liver cirrhosis, HCC can be confidently established in a focal lesion of 1-2 cm by coincident characteristic features in at least two imaging modalities (out of US, CT and MRI), and in a lesion of >2 cm using one imaging modality without needing biopsy confirmation (Barcelona criteria) (7). Imaging should evidence arterial hypervascularization. Adhering to this explains why the diagnosis was not based on histopathology in all patients. This was the case also at several previous PET-studies in HCC (9,10,17,18).

The rationale of using ^11^C-acetate in the diagnosis of HCC instead of FDG at PET examination has been a higher absolute uptake of the primary tumor compared to FDG. This could be confirmed in the current study together with a higher uptake of ^11^C-acetate also compared to the blood activity. The value of this is, however, limited as the tumor uptake versus the surrounding liver tissue is relevant for tumor detection. The paradoxical net effect of our findings is that in the ^11^C-acetate positive PET examinations, the contrast between the tumor activity and the surrounding regular liver activity was lower than the corresponding ratio of the FDG positive tumors. Consequently, the higher tumor uptake of ^11^C-acetate cannot be taken full advantage of in the depiction of primary HCC.

The current study was made in order to allow an optimal assessment of the tracer uptake by the tumor, why? its location was known at the evaluation. Consequently, conclusions regarding the detectability of HCC are restricted. It is possible that some of the HCC’s would have remained undetected at PET examination only with ^11^C-acetate because of the restricted relative uptake together with the irregular activity of the surrounding often cirrhotic tissue. Although not reported, this may have been the same at the previously published studies. The detectability using FDG (30%, 13/44) was lower compared to previously reported figures (Table 6). This cannot be explained outside the ethnical difference of current patients compared previous studies. Our other values were in the same level as the previous reports confirming that adding registration(acquisition?) with 11C-acetate to registration (acquisition?) with FDG at PET examination has an incremental value in the diagnosis of HCC.

An effort was made to analyse if there was any correlation between the tumor differentiation grade and the uptake of the tracers, which has been reported in previous studies. This, however, turned out not feasible because of the restricted number of patients with histopathology together with the small number of poorly and well differentiated tumors causing a sampling bias against these groups.

The analysis of the correlation between the uptake of the two tracers was made because of previous reports of a negative correlation between their uptake at PET examination in HCC (6,10,19). This was only supported by a tendency.

A striking finding was the reciprocal tracer distribution in 4 tumors. One explanation may be regional differences of vascularisation. The uptake of tracers with high extraction rates, like ^11^C-acetate, largely depends on blood flow and shows a strong accumulation in a region with high perfusion compared to a region with low perfusion, while the distribution of an agent with a lower extraction rate, like FDG, depends less on the blood flow (20). Another explanation may be heterogeneity of the tumor. All tumors showing this phenomenon were large, and in a long-standing malignancy different cell clones may change functional properties during growth. Regional variations in the differentiation grade of the same tumor have also been reported in HCC (11). A reciprocal tracer uptake has been reported in a study of the expression of enzymes involved in the intermediary metabolism at HCC (19). That study showed differences of the activity of various enzymes involved in the metabolism of glucose and acetate, as well as in the synthesis of fatty acids between HCC’s accumulating either ^11^C-acetate or FDG. Such a functional heterogeneity may be relevant both for intratumoral differences as well as for differences between various tumors.

## CONCLUSION

The findings confirm that adding registration (acquisition?) with ^11^C-acetate to registration (acquisition?) with FDG at PET examination has an incremental value in the diagnosis of primary HCC. Although not required in patients fulfilling the indirect criteria regularly used in this diagnosis, it should be of complementary value in unclear cases. A higher tumor uptake of ^11^C-acetate cannot be taken full advantage of for tumor detection because of a relatively higher uptake by the surrounding liver tissue which reduces the image contrast and, thereby, the detectability of the tumour. 

Acknowledgments. The authors wish to thank Elisabeth Berg, B.Sc., Karolinska Institutet, for the highly professional statistical analysis. The current address of Dr. Arvidsson is Centre for Minimal Invasive Surgery, Stockholm, Sweden. The current address of Dr. Jersenius is Bayer AB, Solna, Sweden.

## Figures and Tables

**Table 1:**
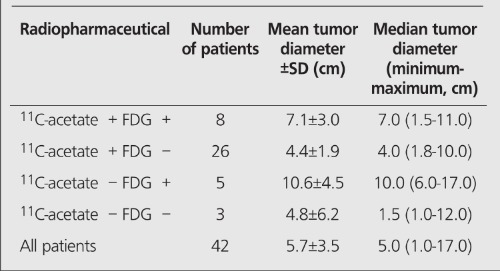
Largest tumor diameter in 42 evaluable patients with HCCvs. positive or negative finding at examinations with ^11^C-acetate andFDG, respectively

**Table 2:**
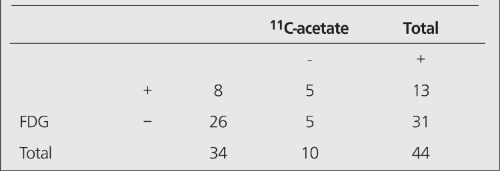
Matrix analysis showing number of positive PET examinationsusing FDG and ^11^C-acetate in 44 patients with hepatocellularcarcinoma (HCC). For statistical analysis (c.f. Results) McNemar’s testwas used

**Table 3:**
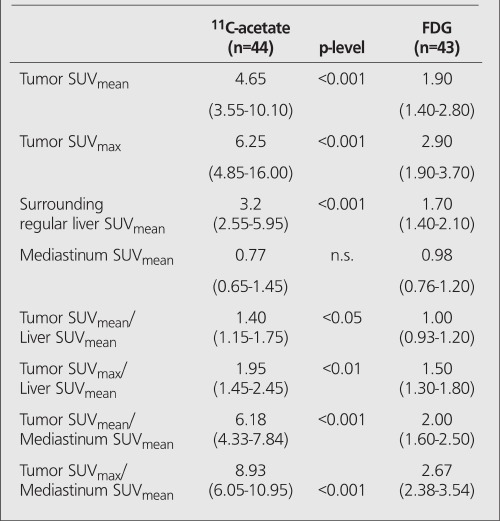
Median tracer uptake and inter-quartile range of the tumorat PET examinations using FDG and ^11^C-acetate in all patients withhepatocellular carcinoma. SUV’s and values vs. mean surroundingliver activity and vs. mean mediastinal blood activity, respectively, areshown

**Table 4:**
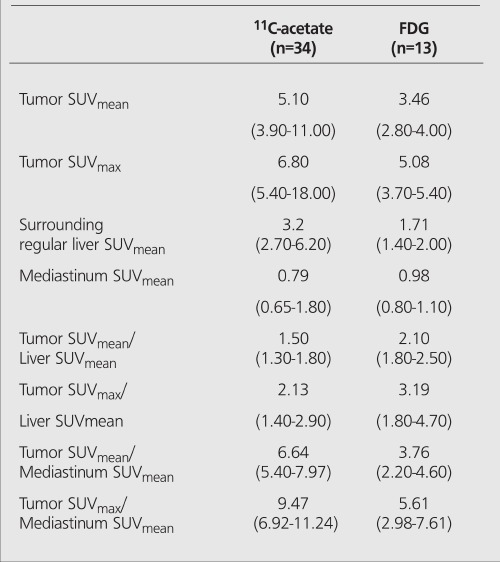
Median tracer uptake and inter-quartile range at PETexaminations in hepatocellular carcinomas being positive using FDGor ^11^C-acetate. SUV’s and values vs. surrounding liver activity and vs.mediastinal blood pool activity, respectively, are shown. Incomparisons between the groups, there are both dependent andindependent observations why? no statistical analyses

**Table 5:**
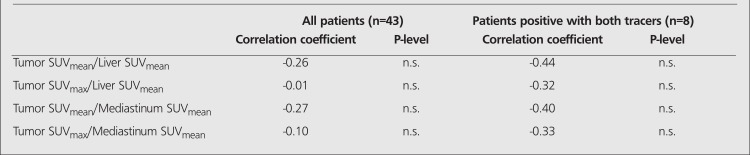
Correlation between the uptake of ^11^C-acetate and of FDG at PET examination in patients with hepatocellular carcinoma (HCC). Allevaluable patients and patients positive with both tracers, respectively, were assessed using Spearman Rank correlation analysis

**Table 6:**
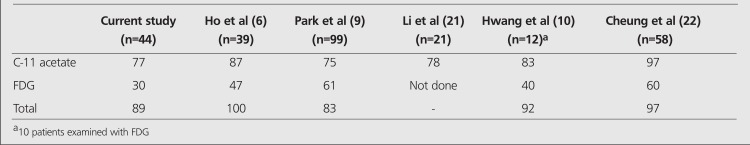
Detectability (percentage) of primary hepatocellular carcinoma (HCC) at PET using FDG and ^11^C-acetate in the current study comparedto previous studies. n=number of patients studied
